# Supplementary material for: Comparative multiomics analysis of cell physiological state after culture in a basket bioreactor
Source: Sci Rep. 2022 Nov 23;12:20161. doi: 10.1038/s41598-022-24687-4 (PMC9686226; doi:10.1038/s41598-022-24687-4)
Supplement: Supplementary file 1 — Supplementary Information 1. [file 41598_2022_24687_MOESM1_ESM.zip › raw data/Transcriptomic raw data/6.Enrichment/2.KEGG/TE1vsTC1/GSEA/gsea_report_for_TC1_1656483128235.html]

Report for TC1 1656483128235 [GSEA]

| GS  follow link to MSigDB | GS DETAILS | SIZE | ES | NES | NOM p-val | FDR q-val | FWER p-val | RANK AT MAX | LEADING EDGE || 1 | RETROGRADE\_ENDOCANNABINOID\_SIGNALING(CSAB04723) | Details ... | 5 | -0.68 | -1.31 | 0.000 | 1.000 | 0.484 | 7179 | tags=100%, list=32%, signal=147% |
| 2 | PHOSPHATIDYLINOSITOL\_SIGNALING\_SYSTEM(CSAB04070) | Details ... | 1 | -0.76 | -1.24 | 0.000 | 1.000 | 0.816 | 5392 | tags=100%, list=24%, signal=132% |
| 3 | PENTOSE\_AND\_GLUCURONATE\_INTERCONVERSIONS(CSAB00040) | Details ... | 1 | -0.91 | -1.19 | 0.000 | 1.000 | 0.816 | 1923 | tags=100%, list=9%, signal=109% |
| 4 | ASCORBATE\_AND\_ALDARATE\_METABOLISM(CSAB00053) | Details ... | 1 | -0.91 | -1.19 | 0.000 | 1.000 | 0.816 | 1923 | tags=100%, list=9%, signal=109% |
| 5 | GLYCOSAMINOGLYCAN\_DEGRADATION(CSAB00531) | Details ... | 1 | -0.91 | -1.19 | 0.000 | 0.863 | 0.816 | 1923 | tags=100%, list=9%, signal=109% |
| 6 | PORPHYRIN\_METABOLISM(CSAB00860) | Details ... | 1 | -0.91 | -1.19 | 0.000 | 0.719 | 0.816 | 1923 | tags=100%, list=9%, signal=109% |
| 7 | DRUG\_METABOLISM\_\_\_OTHER\_ENZYMES(CSAB00983) | Details ... | 1 | -0.91 | -1.19 | 0.000 | 0.617 | 0.816 | 1923 | tags=100%, list=9%, signal=109% |
| 8 | LYSOSOME(CSAB04142) | Details ... | 1 | -0.91 | -1.19 | 0.000 | 0.539 | 0.816 | 1923 | tags=100%, list=9%, signal=109% |
| 9 | RAS\_SIGNALING\_PATHWAY(CSAB04014) | Details ... | 2 | -0.64 | -1.09 | 0.000 | 1.000 | 0.816 | 8052 | tags=100%, list=36%, signal=156% |
| 10 | COMPLEMENT\_AND\_COAGULATION\_CASCADES(CSAB04610) | Details ... | 1 | -0.90 | -1.01 | 0.000 | 1.000 | 1.000 | 2193 | tags=100%, list=10%, signal=111% |
| 11 | PERTUSSIS(CSAB05133) | Details ... | 1 | -0.90 | -1.01 | 0.000 | 1.000 | 1.000 | 2193 | tags=100%, list=10%, signal=111% |
| 12 | RIG\_I\_LIKE\_RECEPTOR\_SIGNALING\_PATHWAY(CSAB04622) | Details ... | 2 | -0.60 | -1.00 | 0.000 | 1.000 | 1.000 | 8916 | tags=100%, list=40%, signal=166% |
| 13 | GLYCOLYSIS\_\_\_GLUCONEOGENESIS(CSAB00010) | Details ... | 1 | -0.84 | -0.99 | 0.329 | 1.000 | 1.000 | 3550 | tags=100%, list=16%, signal=119% |
| 14 | PENTOSE\_PHOSPHATE\_PATHWAY(CSAB00030) | Details ... | 1 | -0.84 | -0.99 | 0.329 | 1.000 | 1.000 | 3550 | tags=100%, list=16%, signal=119% |
| 15 | FRUCTOSE\_AND\_MANNOSE\_METABOLISM(CSAB00051) | Details ... | 1 | -0.84 | -0.99 | 0.329 | 1.000 | 1.000 | 3550 | tags=100%, list=16%, signal=119% |
| 16 | GALACTOSE\_METABOLISM(CSAB00052) | Details ... | 1 | -0.84 | -0.99 | 0.329 | 1.000 | 1.000 | 3550 | tags=100%, list=16%, signal=119% |
| 17 | RNA\_DEGRADATION(CSAB03018) | Details ... | 1 | -0.84 | -0.99 | 0.329 | 1.000 | 1.000 | 3550 | tags=100%, list=16%, signal=119% |
| 18 | HIF\_1\_SIGNALING\_PATHWAY(CSAB04066) | Details ... | 1 | -0.84 | -0.99 | 0.329 | 1.000 | 1.000 | 3550 | tags=100%, list=16%, signal=119% |
| 19 | THYROID\_HORMONE\_SIGNALING\_PATHWAY(CSAB04919) | Details ... | 1 | -0.84 | -0.99 | 0.329 | 1.000 | 1.000 | 3550 | tags=100%, list=16%, signal=119% |
| 20 | GLUCAGON\_SIGNALING\_PATHWAY(CSAB04922) | Details ... | 1 | -0.84 | -0.99 | 0.329 | 1.000 | 1.000 | 3550 | tags=100%, list=16%, signal=119% |
| 21 | CENTRAL\_CARBON\_METABOLISM\_IN\_CANCER(CSAB05230) | Details ... | 1 | -0.84 | -0.99 | 0.329 | 1.000 | 1.000 | 3550 | tags=100%, list=16%, signal=119% |
| 22 | CHOLINE\_METABOLISM\_IN\_CANCER(CSAB05231) | Details ... | 1 | -0.64 | -0.99 | 0.325 | 0.968 | 1.000 | 8166 | tags=100%, list=36%, signal=157% |
| 23 | HEDGEHOG\_SIGNALING\_PATHWAY(CSAB04340) | Details ... | 1 | -0.72 | -0.98 | 0.333 | 1.000 | 1.000 | 6255 | tags=100%, list=28%, signal=139% |
| 24 | NOTCH\_SIGNALING\_PATHWAY(CSAB04330) | Details ... | 1 | -0.74 | -0.97 | 0.332 | 1.000 | 1.000 | 5899 | tags=100%, list=26%, signal=136% |
| 25 | TH1\_AND\_TH2\_CELL\_DIFFERENTIATION(CSAB04658) | Details ... | 1 | -0.74 | -0.97 | 0.332 | 1.000 | 1.000 | 5899 | tags=100%, list=26%, signal=136% |
| 26 | OXIDATIVE\_PHOSPHORYLATION(CSAB00190) | Details ... | 8 | -0.40 | -0.97 | 0.488 | 1.000 | 1.000 | 13464 | tags=100%, list=60%, signal=251% |
| 27 | THERMOGENESIS(CSAB04714) | Details ... | 8 | -0.40 | -0.97 | 0.488 | 1.000 | 1.000 | 13464 | tags=100%, list=60%, signal=251% |
| 28 | ALZHEIMER\_DISEASE(CSAB05010) | Details ... | 8 | -0.40 | -0.97 | 0.488 | 1.000 | 1.000 | 13464 | tags=100%, list=60%, signal=251% |
| 29 | PARKINSON\_DISEASE(CSAB05012) | Details ... | 8 | -0.40 | -0.97 | 0.488 | 0.969 | 1.000 | 13464 | tags=100%, list=60%, signal=251% |
| 30 | AMYOTROPHIC\_LATERAL\_SCLEROSIS(CSAB05014) | Details ... | 8 | -0.40 | -0.97 | 0.488 | 0.937 | 1.000 | 13464 | tags=100%, list=60%, signal=251% |
| 31 | HUNTINGTON\_DISEASE(CSAB05016) | Details ... | 8 | -0.40 | -0.97 | 0.488 | 0.907 | 1.000 | 13464 | tags=100%, list=60%, signal=251% |
| 32 | PRION\_DISEASE(CSAB05020) | Details ... | 8 | -0.40 | -0.97 | 0.488 | 0.878 | 1.000 | 13464 | tags=100%, list=60%, signal=251% |
| 33 | PATHWAYS\_OF\_NEURODEGENERATION\_\_\_MULTIPLE\_DISEASES(CSAB05022) | Details ... | 8 | -0.40 | -0.97 | 0.488 | 0.852 | 1.000 | 13464 | tags=100%, list=60%, signal=251% |
| 34 | CHEMICAL\_CARCINOGENESIS\_\_\_REACTIVE\_OXYGEN\_SPECIES(CSAB05208) | Details ... | 8 | -0.40 | -0.97 | 0.488 | 0.827 | 1.000 | 13464 | tags=100%, list=60%, signal=251% |
| 35 | MALARIA(CSAB05144) | Details ... | 1 | -0.75 | -0.97 | 0.333 | 0.815 | 1.000 | 5598 | tags=100%, list=25%, signal=133% |
| 36 | NEUROTROPHIN\_SIGNALING\_PATHWAY(CSAB04722) | Details ... | 1 | -0.89 | -0.94 | 0.663 | 0.858 | 1.000 | 2475 | tags=100%, list=11%, signal=112% |
| 37 | MEASLES(CSAB05162) | Details ... | 1 | -0.89 | -0.94 | 0.663 | 0.835 | 1.000 | 2475 | tags=100%, list=11%, signal=112% |
| 38 | P53\_SIGNALING\_PATHWAY(CSAB04115) | Details ... | 2 | -0.57 | -0.88 | 0.662 | 0.951 | 1.000 | 9626 | tags=100%, list=43%, signal=175% |
| 39 | DIABETIC\_CARDIOMYOPATHY(CSAB05415) | Details ... | 9 | -0.34 | -0.88 | 0.488 | 0.931 | 1.000 | 14881 | tags=100%, list=66%, signal=298% |
| 40 | AMPK\_SIGNALING\_PATHWAY(CSAB04152) | Details ... | 2 | -0.61 | -0.88 | 0.497 | 0.912 | 1.000 | 8786 | tags=100%, list=39%, signal=165% |
| 41 | OXYTOCIN\_SIGNALING\_PATHWAY(CSAB04921) | Details ... | 1 | -0.61 | -0.82 | 0.662 | 0.987 | 1.000 | 8786 | tags=100%, list=39%, signal=165% |
| 42 | LEUKOCYTE\_TRANSENDOTHELIAL\_MIGRATION(CSAB04670) | Details ... | 1 | -0.53 | -0.81 | 0.333 | 0.968 | 1.000 | 10611 | tags=100%, list=47%, signal=190% |
| 43 | HEPATITIS\_B(CSAB05161) | Details ... | 3 | -0.34 | -0.75 | 0.492 | 0.972 | 1.000 | 14881 | tags=100%, list=66%, signal=298% |
| 44 | CARDIAC\_MUSCLE\_CONTRACTION(CSAB04260) | Details ... | 3 | -0.40 | -0.74 | 0.658 | 0.957 | 1.000 | 13464 | tags=100%, list=60%, signal=251% |
| 45 | NON\_ALCOHOLIC\_FATTY\_LIVER\_DISEASE(CSAB04932) | Details ... | 3 | -0.40 | -0.74 | 0.658 | 0.936 | 1.000 | 13464 | tags=100%, list=60%, signal=251% |
Table: Gene sets enriched in phenotype **TC1 (2 samples) **[plain text format]****
